# Innate and Adaptive Responses of Intratumoral Immunotherapy with Endosomal Toll-Like Receptor Agonists

**DOI:** 10.3390/biomedicines10071590

**Published:** 2022-07-04

**Authors:** Fernando Torres Andón, Sergio Leon, Aldo Ummarino, Esther Redin, Paola Allavena, Diego Serrano, Clément Anfray, Alfonso Calvo

**Affiliations:** 1 Center for Research in Molecular Medicine and Chronic Diseases, Universidade de Santiago de Compostela, 15706 Santiago de Compostela, Spain; fernando.torres.andon@usc.es; 2 IRCCS Humanitas Research Hospital, 20089 Rozzano, Italy; paola.allavena@humanitasresearch.it; 3 Program in Solid Tumors, Center for Applied Medical Research (CIMA), Department of Pathology and Histology, University of Navarra, 31008 Pamplona, Spain; sleon.1@alumni.unav.es (S.L.); eredin@alumni.unav.es (E.R.); dserrano@unav.es (D.S.); 4 Laboratory of Cellular Immunology, Humanitas University, 20089 Pieve Emanuele, Italy; aldo.ummarino@hunimed.eu (A.U.); clement.anfray@humanitasresearch.it (C.A.); 5 Centro de Investigación Biomédica en Red Cáncer (CIBERONC), Avenida Monforte de Lemos, 3-5, 28029 Madrid, Spain; 6 Navarra Institute for Health Research (IdiSNA), C/Irunlarrea 3, 31008 Pamplona, Spain

**Keywords:** toll-like receptors, TLR agonists, intratumoral administration, antitumoral immunotherapy, tumor associated macrophages

## Abstract

Toll-like receptors (TLRs) are natural initial triggers of innate and adaptive immune responses. With the advent of cancer immunotherapy, nucleic acids engineered as ligands of endosomal TLRs have been investigated for the treatment of solid tumors. Despite promising results, their systemic administration, similarly to other immunotherapies, raises safety issues. To overcome these problems, recent studies have applied the direct injection of endosomal TLR agonists in the tumor and/or draining lymph nodes, achieving high local drug exposure and strong antitumor response. Importantly, intratumoral delivery of TLR agonists showed powerful effects not only against the injected tumors but also often against uninjected lesions (abscopal effects), resulting in some cases in cure and antitumoral immunological memory. Herein, we describe the structure and function of TLRs and their role in the tumor microenvironment. Then, we provide our vision on the potential of intratumor versus systemic delivery or vaccination approaches using TLR agonists, also considering the use of nanoparticles to improve their targeting properties. Finally, we collect the preclinical and clinical studies applying intratumoral injection of TLR agonists as monotherapies or in combination with: (a) other TLR or STING agonists; (b) other immunotherapies; (c) radiotherapy or chemotherapy; (d) targeted therapies.

## 1. Introduction

Intratumoral (i.t.) administration of immunotherapy has emerged as an effective therapeutic approach against solid tumors. The actual portfolio of immunotherapies applied for the treatment of cancer is led by immune checkpoint inhibitors (i.e., PD-1 or CTLA-4) and adoptive transfer of chimeric antigen receptor (CAR) T cells [1,2,3], and followed by other treatments in the form of cytokines (i.e., IL-2 or IL-12), oncolytic viruses (i.e., T-VEC), other monoclonal antibodies, or small drugs targeting immune cells (i.e., CD40, CD47, or CSF-1R) [4,5]. All these treatments have been tested intravenously, showing good antitumoral activity often limited by systemic inflammation and autoimmune-like reactions [6]. However, their systemic administration also showed poor biodistribution and pharmacokinetic profiles as well as difficulties in reaching the target cells in the tumor microenvironment (TME). To address this issue, basic and clinical researchers have tested the i.t. administration of these immunotherapies, with the goal of limiting systemic immunotoxicity while achieving strong antitumor response [7,8].

Among the variety of pharmacological strategies to stimulate the immune system in the TME, the i.t. administration of toll-like receptor (TLR) agonists might be the simplest and most feasible approach for clinical translation [9]. Natural and synthetic agonists of endosomal TLRs are administered to activate innate and adaptive immune cells in the TME, triggering antitumoral responses. On the contrary, the stimulation of TLRs in tumor cells has been related with protumoral functions. Moreover, in some types of cancer, such as breast or lung, the comparison between the tumor and the normal tissue revealed the overexpression of certain TLRs, which was associated with an adverse prognosis and higher probability of metastasis [10]. These observations highlight the relevance of controlling the dose and time of administration of TLR agonists for the treatment of cancer. They also emphasize the relevance of recent nanotechnological approaches intended to improve their ability to reach the right tissular and cellular target [11,12].

Combinations of TLR agonists (*ag*) or adding other therapeutic modalities, such as radiotherapy, chemotherapy, or other immunotherapies, have shown in many cases synergistic antitumoral activity without increasing adverse effects. In the last years, the number of investigations following this line of research is increasing exponentially. The possibilities for i.t. combination therapies are high and further studies are needed to understand the mechanisms activated at local and systemic levels in each patient. The clinical implementation of this strategy may be inadequate for some patients, while others, i.e., those with accessible metastatic lesions, may greatly benefit from this therapeutic modality. In fact, ongoing clinical trials are using this approach in patients with aggressive solid tumors.

## 2. Structure and Function of Endosomal TLRs: Triggers of Innate and Adaptive Immune Responses

The discovery of TLRs and their role in innate immunity, alongside dendritic cells in adaptive immunity, was awarded in 2011 with the Nobel Prize in Physiology and Medicine [13]. TLRs are type I transmembrane glycoproteins constituted by an extracellular domain of leucine rich repeats (LRR), a single transmembrane helix, and an intracellular toll-like/interleukin-1 receptor (TIR) domain in the cytosol. In humans, 10 TLRs have been identified, which are classified into extracellular (TLR1, TLR2, TLR5, TLR6, and TLR10 on the cell surface) or intracellular (TLR3, TLR7, TLR8, TLR9 on endosomal/lysosomal membranes), while TLR4 is found in both the plasma membrane and endosomal vesicles [14]. TLRs are specialized in the recognition of foreign or host danger signals in the form of sugars, lipoproteins, or nucleic acid residues, with the purpose of triggering a defensive immune response. Depending on their origin, TLR*ag* may be classified into three categories: (1) exogenous pathogen-associated molecular patterns (PAMPs) released from bacteria or viruses that induce immune responses to fight the infectious disease; (2) endogenous damage-associated molecular patterns (DAMPs) produced and released by damaged cells in case of cellular stress/tissue injury, such as cancer; (3) synthetic TLR*ag*, designed for research and therapeutic purposes [15]. Of note, stimulation of TLRs by PAMPs or DAMPs activates a defensive immune response that results in the clearance of the pathogen/injury and tissue repair. However, uncontrolled TLR activation due to chronic infection, autoimmune diseases, or cancer may aggravate the disease and even result in the death of the host. Although more investigations are needed, it has been speculated that key factors that determine the outcome may depend on: (a) the type of TLR; (b) level of expression; (c) type of cell and tissue target; and (d) dose and time of interaction with the receptor (see Section 3, for relevant observations in the TME).

**Figure 1:**
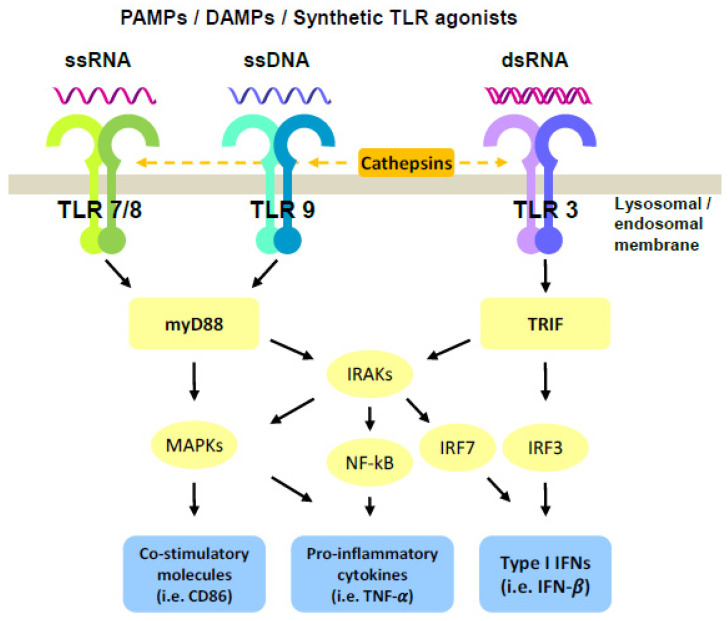
Schematic representation of endosomal TLR activation pathways. Upon ligation of endosomal TLRs, stimulation of myD88 or TRIF signaling results in the activation of co-stimulatory molecules, secretion of pro-inflammatory cytokines and Type I IFNs responses. Furthermore, combination treatment with TLR agonists triggers a synergistic induction of NF-kB, IRFs, or MAPKs pathways, leading to enhanced outcomes.

Upon ligand recognition by the LRRs, the intracellular TIR domain recruits the myeloid differentiation primary response gene 88 (MyD88) in the case of all TLRs, except for TLR3, leading to the activation of the interleukin-1 receptor associated kinases (IRAKs), nuclear factor kB (NF-kB), and mitogen-activated protein kinase (MAPK) pathways, mainly resulting in the production of pro-inflammatory cytokines (Figure 1). In contrast, TRIF is exclusively recruited by TLR3 to activate IFN regulatory factor (IRF) family members, which then induce the expression of genes encoding type I IFNs [16] (Figure 1). Extracellular TLRs are mainly involved in recognition of microbial-PAMPs, leading to secretion of cytokines. On the other hand, activation of endosomal TLRs requires not only ligand interaction but also cleavage of their ectodomains by cathepsins as a prerequisite for signal transduction [17]. Endosomal TLRs are specialized in the recognition of nucleic acids, such as double-stranded (ds) RNA (TLR3), single-stranded (ss) RNA (TLR7 and TLR8), or unmethylated CpG containing ssDNA (TLR9) (Figure 1). Free nucleoside binding occurs within the dimerization interface, whereas RNA or DNA binding is thought to reinforce these interactions and stabilize dimer formation, thus increasing the activity of endosomal TLRs [18]. In the case of TLR3, high-affinity receptor–ligand interaction was observed at acidic pH [19]. Interestingly, affinity of TLR3 and TLR9 for their ligands occurs in the nanomolar concentration, while it needs higher concentrations for TLR7 and TLR8 [15]. It was hypothesized that these complex mechanisms of endosomal TLR activation, which must be in place to maximize the distinction between self- and non-self-nucleic acids, could be potentially exploited to optimize therapeutic responses by TLR*ag* for the treatment of cancer.

## 3. Role of Endosomal TLRs on Tumor Cells and the Tumor Microenvironment

Several clinical studies have evaluated the expression of TLRs as diagnostic/prognostic indicators in the bulk of solid tumors, although without considering the cellular heterogeneity of the TME. These studies showed that expression of TLRs in tumors depends on the type and stage of cancer and differs from one patient to another. For example, higher levels of TLR7/8 and 9 were found in non-small cell lung carcinoma (NSCLC) than in normal lung tissue [20,21]. A meta-analysis in various cancer types found that higher TLR7 expression predicts poor survival and bad prognosis [22]. Expression of TLR9 was positively associated with tumor size and stage in esophageal carcinomas and cervical squamous cell carcinoma [23,24]. Certain TLR polymorphisms have also been associated with increased risk of cancer [10]. Breast carcinomas with high TLR3 expression in tumor cells were associated with worse prognosis and higher probability of metastasis [25]. Interestingly, another study found that activation of TLR3 prior to metastasis inhibited migration of cancer cells, while its activation during metastasis enhanced their migration [26].

**Figure 2:**
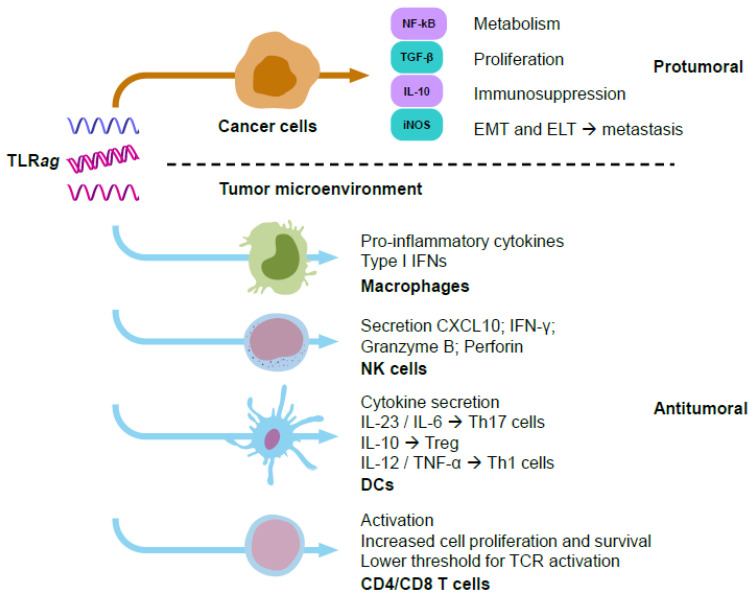
Schematic representation of responses triggered by TLR*ag* in cancer cells (mainly with a protumoral effect) versus immune cells (mainly with antitumoral immune responses). Many experimental studies have shown the activation of protumoral functions by TLR agonists on cancer cells, such as increasing their metabolism and proliferation, epithelial–mesenchymal/leucocytic transition, metastasis, and immunosuppression. On the other hand, TLR agonists activate antitumoral functions on immune cells, such as secretion of pro-inflammatory cytokines, Type I IFNs, increase in levels of perforins or granzyme B, as well as proliferation and recruitment of immune cells to fight against cancer cells.

TLR signaling in cancer cells can induce protumoral effects as a result of: (i) activation of NF-kB, which increases their metabolism and proliferation; (ii) production of immunosuppressive mediators, such as IL-10, TGF-β, and iNOS; (iii) promotion of epithelial-to-mesenchymal transition (EMT) or epithelial-to-leucocytic transition (ELT). These effects would lead to immune evasion and metastasis [27] (Figure 2). Other contradictory observations upon TLR activation in cancer cells have been reported. TLR3 stimulation promoted metabolic reprograming in cancer cells, switching oxidative phosphorylation to anabolic glycolysis (through upregulation of HIF-1α) and enabling better adaptation to hypoxia and oxidative stress in the TME [28]. Another study showed that TLR3 stimulation with poly(I:C) activated the NF-kB pathway without affecting cell viability. However, stimulation of TLR9 with CpG nucleotides (*nt*) promoted cell proliferation and inhibited apoptosis, which ultimately decreased the antitumoral efficacy of adriamycin [29,30]. On the contrary, it has been reported that poly(I:C), R837 (imiquimod^®^, TLR*ag*), and CpG*nt* can induce tumor cell apoptosis and increase sensitivity to radiotherapy [31]. Several studies focused on the protumoral role of TLR7 activation have been reported. Dajon et al. found that TLR7 stimulation increased vimentin and reduced E-cadherin expression in NSCLC cells, inducing an EMT phenotype and metastasis [32]. Others demonstrated that TLR7 stimulation increased proliferation and tumor growth in a pancreatic cancer model [33]. Furthermore, TLR7 stimulation was related to chemoresistance towards 5-fluorouracil in PANC1 cells [33], or to cisplatin combined with gemcitabine or vinorelbine in NSCLC patients [21]. Stimulation of TLR7 and TLR8 with R848 (resiquimod^®^) led to activation of NF-kB, COX2, and BCL-2 in human NSCLC cells, thus promoting proliferation [34]. TLR8 signaling reversed tumor-induced T-cell senescence by blocking cAMP production in tumor cells [35].

In the TME, TLRs can be expressed by multiple immune cells, including monocytes, macrophages, dendritic cells, B cells, T cells, mast cells, and natural killer (NK) cells, as well as by non-immune cells, such as epithelial cells, fibroblasts, and cancer cells [36]. Contrary to cancer cells, TLR signaling in immune cells has mainly been associated with antitumor effects controlled by their immunostimulation and maturation, ultimately leading to cytokine secretion. This triggers pro-inflammatory responses and recruits more immune cells to fight against the tumor (Figure 2). Among the different immune cell populations, tumor-associated macrophages (TAMs) are the most prevalent in most solid tumors, playing a key immunosuppressive role that limits the ability of the immune system to fight cancer [37]. Of note, recent experimental evidence has demonstrated the ability of TLR*ag* to reprogram TAMs into M1-like macrophages with immunostimulatory, phagocytic, and cytotoxic activities towards cancer cells [4]. Basic and clinical studies demonstrating the activation of immune cells by TLR*ag* are provided in the next sections. Overall, TLR stimulation in cancer cells has been mainly associated to protumoral activity, while TLR stimulation in immune cells preferentially leads to the activation of innate and adaptive antitumoral and proinflammatory responses in the TME (Figure 2).

## 4. Preclinical and Clinical Use of TLR (3, 7/8, 9) Agonists in Cancer Treatment

The pharmacological use of TLR*ag* holds great promise for cancer treatment. Notwithstanding, at present, only three TLR*ag* are approved by the FDA for use in patients: the bacillus Calmette-Guérin (BCG) (which acts as agonist of TLR2, TLR4, and TLR9), monophosphoryl lipid A (MPL) (agonist of TLR2 and TLR4), and imiquimod (TLR7*ag*). BCG, prepared from attenuated strains of *Mycobacterium bovis*, is the most widely used vaccine worldwide and has been used to prevent tuberculosis for more than a century [38]. However, oncological clinical trials were only successful against non-muscle-invasive bladder cancer [39]. MPL, chemically derived from *Salmonella minnesota* LPS, has been approved by the FDA as a cervical cancer vaccine, thanks to its immunostimulatory activity and lack of toxicity. Nonetheless, its limited antitumoral activity did not show successful results in other types of cancer [9,40]. Imidazoquinoline derivatives, such as imiquimod, were synthetically developed as antivirals and approved by the FDA in 1999 for the topical treatment of genital warts and actinic keratosis. At that time, the mechanism of action of these drugs had not been described, and later discoveries on the activation of TLRs and acute inflammation and type I INF responses introduced these agents into cancer studies. In 2004, the FDA approved imiquimod (TLR7*ag*) for the treatment of basal cell carcinomas [9]. Later, other TLR7/8 analogs such as resiquimod were developed, showing higher activity in preclinical settings, but failing to prove clinical benefit against genital herpes and hepatitis C [41]. In 2011, clinical trials in hematological neoplasias and solid tumors showed controversial results related to poor antitumoral activity, and immunotoxic effects, including: fever, fatigue, nausea, and cytokine release syndrome [42].

**Figure 3:**
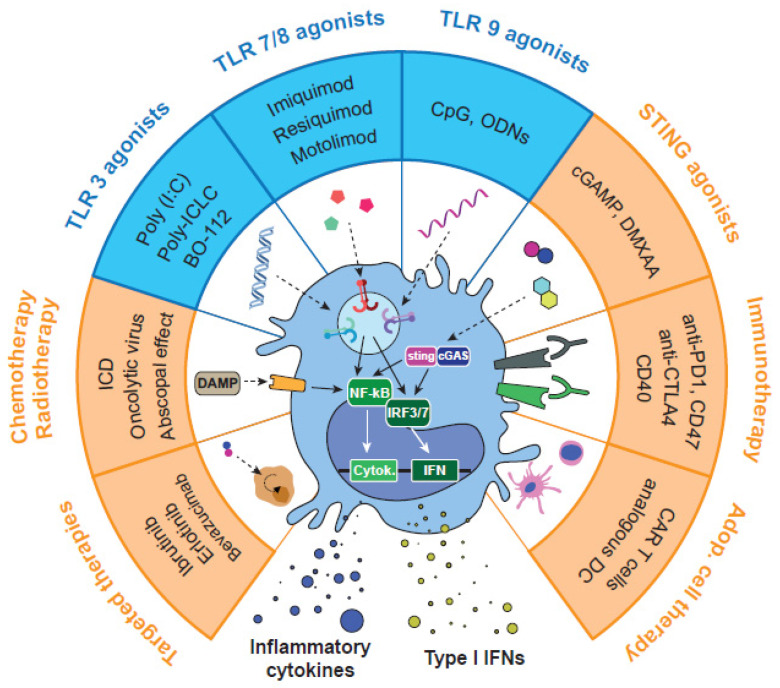
Pharmacological strategies for the activation of endosomal TLRs and combinations with other therapeutic approaches. TLR*ag* alone or in combination have demonstrated the ability to reprogram TAMs towards M1-like antitumor macrophages. This pharmacological approach has been tested in preclinical and clinical studies with other immunotherapies, such as STING agonists, immune checkpoint inhibitors, or adoptive cell therapy. Other combinations include chemotherapy, oncolytic viruses, or radiotherapy, which can kill cancer cells and trigger immunostimulatory responses. Finally, some studies have used combinations with targeted therapy, epigenetic drugs, or metabolic reprogramming drugs.

In our view, a more profound knowledge of TLR signaling and exploration of potential synergies using TLR*ag* in combination with other drugs is highly needed. In the last decade, numerous preclinical and clinical studies have tested endosomal TLR3/7/8/9 agonists against cancer, such as: poly(I:C), poly-ICLC, CpG*nt,* resiquimod, motolimod, MEDI9191, NKTR-262, and LHC165, among others [43,44,45] (Figure 3). According to the literature (summarized in previous sections), activation of endosomal vs. extracellular TLRs may present advantages in terms of specificity and higher activation of immune responses. Furthermore, activation of TLRs in the appropriate immune cells (i.e., while avoiding their activation in cancer cells), is certainly desirable for antitumoral activity. Most TLR*ag* used as single therapy to stimulate the immune system were not successful in clinical trials, due to low efficacy or severe side effects. Of note, repeated activation of TLRs has been related to immunotolerance, as well as with a shift in the immune system towards a pro-resolution and anti-inflammatory activity, which supports tumor growth [46]. On the bright side, the most recent work applying combinatorial strategies (reviewed in the next sections) has demonstrated that their appropriate application to certain types of tumors leads to potent, safe, and prolonged antitumor immune responses. Thus, controlling posology (dose and time), route of administration, and biodistribution of these drugs results in foremost importance for their activity. With this purpose, TLR*ag* are being tested through a variety of administration routes: intravenous, intratumoral, subcutaneous, topical, or oral [47]. Among them, local delivery by intratumoral (i.t.) administration has shown the best antitumoral efficacy with low toxicity, not only in the primary tumor but also in distant metastasis. In parallel, the systemic intravenous (i.v.) administration of advanced pharmaceutical formulations have demonstrated the ability to limit drug clearance, reduce toxicity, enhance tumor accumulation, and improve the delivery of the TLR*ag* at the right location.

### 4.1. Intratumor and Systemic Delivery for Cancer Treatment and Vaccination Approaches: Role of Drug Conjugates and Nanoparticle Formulations

TLR*ag* have been investigated as anticancer drugs, usually in combinations, mainly upon i.t. or i.v. administration, or subcutaneously (s.c.) as vaccines in the proximity of the tumor or lymph nodes. Due to important advances in surgical, interventional, and even robot-assisted techniques allowing precise and relatively easy access to almost any malignant tissue, local administration of drugs is gaining enthusiasm [48,49]. In the old days, local intravesical instillation of BCG or topical administration of imiquimod represented particular solutions for very specific types of cancer in the bladder and skin, respectively. More recently, the topical administration of resiquimod in cutaneous T-cell lymphoma showed improvement of lesions in 75% of patients with minor adverse effects [50]. Lately, administration of TLR3/7/8/9*ag* inside the tumor or even in the metastatic lesions has been tested, showing impressive local and systemic antitumoral efficacy with reduced or no toxicity [51]. The i.t. injection of these TLR*ag* triggers acute pro-inflammatory responses in the TME, destroying cancer cells. This activity is accompanied by “in situ vaccination”, which involves the release of antigens from the dead cells and subsequent presentation to the antigen-presenting cells (APCs). This process leads to the recognition and elimination of cancer cells in non-injected locations (abscopal effect) [7]. Many of these experiments testing local administration of TLR*ag* involved their conjugation or nanoencapsulation [11].

For vaccination purposes, drugs simulating PAMPs have been tested as adjuvants due to their capacity to stimulate cell-mediated immunity, mainly by direct targeting of DCs. While the first FDA approved adjuvant molecule was MPL [52], nowadays, agonists of endosomal TLRs are preferentially used in numerous cancer vaccination trials. The administration of poly(I:C), probably the most paradigmatic TLR*ag* adjuvant in different antigen formulations, was highly effective in several preclinical models, irrespective of the route of administration [53]. Poly(I:C) and poly-ICLC (hiltonol^®^) activate DCs and NK cells and stimulate cross-priming. Furthermore, their intra- or peritumor injection in mouse models with established tumors showed high antitumoral activity. However, attempts to translate poly(I:C)-based strategies to clinical practice were not successful, probably because of its short half-life [54]. As a matter of fact, poly-ICLC, a more stable formulation, was more effective but highly toxic [53]. As an alternative approach, Han et al. developed a PLGA-NP system encapsulating both ovalbumin (OVA) and poly(I:C) that was injected subcutaneously in mice with lymphoma and lung cancer implanted tumors. Such treatment increased the efficiency of intracellular drug delivery into DCs and promoted DC maturation and antigen-specific cross-presentation [55]. Activation of DCs by i.t. injection of poly(A:U), another TLR3*ag*, was observed in the murine B16.F10-OVA melanoma model, with an increase in CD8^+^ T cell infiltration and decrease in IL-10-producing M2-like macrophages [56]. TLR7/8*ag* have also been used as vaccine adjuvants, with positive results in most cases. Conjugation of TLR7/8*ag* with large antigen-containing particles led to more effective CD8^+^ T cell responses and higher uptake by DCs compared to the use of the single antigens. Other nanotechnological approaches have been developed for the administration of TLR7/8*ag* [47]. Similarly, CpG*nt*, which activate TLR9 in plasmacytoid dendritic cells (pDCs) and cytotoxic T lymphocytes (CTLs), have been tested individually or in combination with cancer vaccines, but have not reached clinical approval yet. To improve their short half-life and low immunostimulatory activity, numerous CpG formulations are under clinical investigation [45].

In non-vaccine settings, systemic administration of TLR-formulations include also nanoparticles (NPs) prepared by PEGylation, conjugation with albumin, phospholipids, lipid or polymeric nanocomplexes, and encapsulation into nanocapsules, but also conjugation with antibodies or other ligands [11,57]. For example, intravenous administration of cyclodextrin NPs carrying resiquimod has demonstrated antitumor efficacy in MC38 colon cancer and B16.F10 melanoma mouse models [58]. Other studies have shown antitumor and antimetastatic activity using resiquimod-encapsulated poly(2-oxazoline) NPs in an orthotopic model of NSCLC, an effect that was mediated by Ly6C^+^ monocytes and CD8^+^ T lymphocytes [59]. Other NP-formulations enclosing resiquimod have been successfully tested in mice, showing a significant improvement in the targeting of APCs and anticancer response. Bahmani et al. demonstrated that i.t. delivery of platelet-cloaked NPs loaded with resiquimod achieved complete tumor regression in the MC38 colon carcinoma model, also preventing tumor growth after re-challenge. This strategy maximized local immune activation [60]. An insightful study carried out by Hayashi et al. studied the therapeutic effect of TLR7*ag* on melanoma, using the B16cOVA model [61]. The TLR7*ag* 1V199 directly injected into the tumor significantly inhibited tumor growth when low repeated doses were used, whereas a single high dose was ineffective. The phospholipid- or PEG-conjugated 1V199 variants 1V270 and 1V285 (respectively) were also tested. Treatment with 1V285 was not effective, whereas 1V270 was highly efficacious, prolonging mice survival compared to the unconjugated variant. Another interesting study conducted by Thomas et al. explored the particular TME pathways activated by i.t. injection of TLR3, TLR7, or TLR9 agonists in B16.F10 melanoma-bearing mice [62]. This model was first characterized by single-cell RNAseq to study TLR expression in the different TME populations, showing that expression of TLR3 was restricted to classical DCs (cDCs), whereas TLR7 was highly expressed across TAMs and pDCs. TLR9 was less expressed, and only found in pDCs and some monocyte/macrophage subsets. TLR3 stimulation induced the expression of type-I INF and antigen presentation signatures. It also caused a profound reduction in the number of infiltrating Tregs and elicited CD8^+^ T cell activation, while TLR7 stimulation decreased the number of TAMs [62]. The authors hypothesized that a synergistic activation of this particular signaling pathway with combinations of TLR*ag* may significantly increase the antitumoral efficacy.

### 4.2. Combinations with Other TLR Agonists or STING Agonists

Recent investigations have unveiled the synergistic antitumoral activity of certain TLR-TLR and TLR-STING (cGAS/stimulator of interferon genes) agonist combinations (Figure 3). We have recently found the superior therapeutic efficacy of poly(I:C) combined with resiquimod, versus the combination with imiquimod, or any of these TLR*ag* as monotherapy, using immunocompetent murine models of lung cancer and fibrosarcoma [63]. Our comprehensive analysis of the TME demonstrated that antitumoral activity was mainly driven by macrophage reprogramming towards M1-like antitumor effector cells, which promoted the activation of innate and adaptive immune responses against the cancer cells. Although we did not find significant involvement of NK cells in our murine models, other researchers have demonstrated their key antitumor contribution upon TLR3 or TLR7/8 activation. Such an effect was mediated by the production of IFN-γ, CXCL10, granzyme B, and perforin in melanoma and HNSCC [64,65,66]. Other authors demonstrated that TLR3 and TLR7 agonists increased the expression of CD54 in γδ-T cells and lysis of pancreatic cancer cells [67]. We found that the poly(I:C)+ resiquimod combination required theactivity of CD4^+^ and CD8^+^ T cells for an effective antitumoral response in a lung cancer model [63]. Several studies also performed combinations of TLR3*ag* or TLR7/8*ag* with CpG*nt* (TLR9*ag*) by i.t. injections. In a murine glioma model, poly(I:C) and CpG*nt* were administered i.p. and i.t., respectively. This combination inhibited tumor growth and improved the median survival. The treatment efficacy was attributed to the activation of an antitumor phenotype of microglia, which triggered the release of pro-inflammatory cytokines, in particular IFN-β, increasing motility and phagocytic activity of these cells to directly kill glioma cells. Furthermore, the antitumoral efficacy of this TLR combination was enhanced by addition of CD47 blocking antibodies [68]. Others tested the co-delivery of TLR7/8*ag* and TLR9*ag* (3M-052 and CpG*nt*, respectively) in a subcutaneous colon carcinoma mouse model. Again, within the TME, this combination of TLR*ag* upregulated the expression of Th1 cytokines, reduced the number of immunosuppressive tumor resident myeloid-derived suppressor cells (MDSCs), and increased the accumulation of NK cells and CD8^+^ T lymphocytes, leading to a strong and long-lasting antitumoral immune response [69]. Another group combined resiquimod with the alarmin HMGB1 (TLR4 ligand) and cyclophosphamide (small drug with cytotoxic and immunosuppressive activity) i.t. in the CT26 model. This combined treatment increased the infiltration of T cells in the tumor, as well as the activation and homing of tumor-infiltrating DCs to the draining lymph node As a result, eradication of large established tumors and resistance to re-challenge was observed [70]. In a recent study, Manna et al. developed a new supramolecular formulation of the covalently linked TLR*ag* Pam2CSK4C and azide (TLR2/6 and TLR7/8 agonists, respectively). In the B16.F10 melanoma model, this new multicomponent TLR*ag* assembly induced CD8^+^ T cell and NK cell antitumor responses, inhibited tumor growth, and reduced adverse effects [71].

TLR*ag*-TLR*ag* combinations have also been tested as adjuvants to improve the efficacy of new cancer vaccines. A recent report by Gondan et al. evaluated poly(I:C) plus imiquimod in the B16.F10(OVA) melanoma model, using zinc-doped iron oxide magnetic NP-loaded phospholipid micelles for vaccination purposes (injected s.c.). They observed a synergistic activation of an antitumor immune response and direct killing of cancer cells, leading to protection against tumor development, but also strong therapeutic activity in established tumors [72]. Da Silva et al. showed that i.t. co-delivery of poly(I:C) + resiquimod + CCL20 (MIP3a) using biodegradable polymeric NPs had profound antitumor effects in the context of peptide vaccination in two lymphoma models. In one of the models, large tumors were rejected after application of this therapy [73].

Regarding TLR*ag*-STING*ag* combinations in mouse models of lymphoma and melanoma, antitumoral activity was achieved with i.t. injections of the TLR9*ag* K3 CpG*nt* and the STING*ag* cGAMP. Using in vitro cultures of human and mouse PBMCs, the authors showed that K3 CpG*nt* and cGAMP induced high production of type-I IFNs and IL-12, secreted from pDCs and macrophages. This triggered a synergistic activation of NK cells, resulting in high production of IFN-γ and activation of CD8^+^ T cell response in vivo. This mechanism was validated in experiments with RAG2 KO mice (lacking CD8^+^ T cells), where the antitumor effect of the combination was lost [74]. In a recent study, the TLR3*ag* BO-112 (a poly(I:C)-nanocomplex) has been combined with the STING*ag* 5,6-dimethylxanthenone-4-acetic acid (DMXAA) in subcutaneous mouse models of colon cancer and melanoma, resulting in strong antitumoral activity. In the MC38 colon cancer model, the combination of BO-112 and DMXAA showed an abscopal effect in contralateral non-injected tumors, while none of the single drugs had such an activity. Interestingly, this abscopal effect was preserved in a third non-injected tumor after the inoculation of both drugs in separate tumors. Their results also suggest that a strong CD8^+^ T cell response is responsible for the antitumor activity, caused by DC-mediated cross-priming and secretion of type I IFNs in response to the combination [75]. The main endosomal TLR agonists, their biological and antitumor effects, and examples of combinations of TLR-TLR*ag* and TLR-STING*ag* can be found in Table 1.

### 4.3. Combination of TLR Agonists with Other Immunotherapies or Adoptive Cell Therapy

Combination of TLR*ag* with immune checkpoint inhibitors (ICI) or other immunotherapy modalities is currently an area of intense research (Figure 3). One of the first TLR3-based combinations evaluated was poly(I:C) + anti-CD40 + OVA in a lymphoma model. This combination protected 94% of mice against tumor development. However, in the absence of OVA, no response was observed [76]. In another preclinical study, ARNAX (TLR3*ag*) overcame anti-PD-L1 resistance and led to tumor regression in vivo by increasing the levels of CD11c^+^ cells [77]. In the clinic, three derivatives of poly(I:C) have been tested in combination with other immunotherapies: BO-112 (poly(I:C)-nanocomplexes), rintatolimod (poly I:C12U), and hiltonol (poly-ICLC). In a phase I clinical trial involving 28 anti-PD-1-resistant patients with solid tumors, i.t. administration of BO-112 in combination with nivolumab or pembrolizumab resulted in three partial responses and 10 stable diseases, showing good tolerability [78]. However, a phase II trial combining pembrolizumab and hiltonol showed no clinical benefit in metastatic mismatch repair-proficient colon cancer patients, where only 8.3% objective response rates (ORR) were found (NCT02834052). Combination of hiltonol with autologous DCs in patients with metastatic or unresectable pancreatic cancer was safe, resulting in stable disease in four patients out of eight [79]. Other clinical trials combining TLR3*ag* with immunotherapy are currently ongoing. Among these, combination of rintatolimod and pembrolizumab is being explored in refractory, metastatic, or unresectable colorectal cancer (NCT04119830). Poly-ICLC + anti-CD40 is also being tested in melanoma patients (NCT04364230). Imiquimod + anti-PD-1 is being studied in a wide variety of solid tumors, including patients with melanoma, breast, NSCLC, SCLC, ovarian, gastric, and hepatocellular cancers (NCT04116320).

The TLR7*ag* 1V270 (i.t.) in combination with anti-PD-1 showed potent tumor suppressive effects by increasing the M1/M2 macrophage ratio and the release of IFN-γ by CD8^+^ T cells in the SCC7 model of HNSCC [80]. Nishii et al. found in CT-26-bearing mice that resiquimod reverted the resistance to anti-PD-L1 by recruiting CD8^+^ T cells and reducing the number of Tregs. However, they also observed in the SCCVII oral squamous carcinoma model that resiquimod did not improve the response to anti-PD-L1 [81]. In another study, imiquimod upregulated the co-stimulatory immune checkpoint OX40 in hepatocellular carcinoma, and its combination with OX40-agonist suppressed tumor growth and induced long-term protection [82]. 1V270 (i.t. injected) has also been combined with systemic IL-2 in a melanoma model, showing improved survival. Such an effect was associated with enhanced CD8^+^ T lymphocyte responses [61]. Other combinations to inhibit immunosuppressive signals in the TME have been explored to enhance the antitumor effect of TLR7/8*ag*. For instance, it was observed that i.t. administration of R837 (imiquimod) significantly increased the expression of the immunosuppressive signals IDO and iNOS in tumor-draining lymph nodes. R837 combined with inhibitors of IDO or iNOS enhanced the therapeutic efficacy via the increase of Th1 immune responses [83,84].

Use of 3M-052 in addition to CpG*nt* has shown impressive effects in preclinical studies of colon cancer, causing tumor regression and developing a longer T cell memory, when compared to single treatments. Mechanistically, this therapy increased the levels of cytotoxic T cells and NK cells [69]. In this same malignancy, NP-based TLR7*ag* potentiated the response to anti-PD-1 + anti-CTLA-4 and induced 60% tumor regressions [85]. In the B16.F10 melanoma model, local administration of the TLR9*ag* ODN1826, in addition to anti-CLTLA-4, resulted in 44% tumor regressions in vivo [86]. However, TLR9*ag* in combination with immunotherapy has not been explored in depth. Sato-Kaneko et al. demonstrated that i.t. administration of SD-101 synergized with anti-PD-1 in preclinical models of HNSCC [80]. This therapeutic combination was explored in a clinical trial and the results showed 24% ORR, with 2 complete and 10 partial regressions [87]. A similar combination was also evaluated in patients with unresectable or metastatic malignant melanoma in a phase Ib trial. Results showed stimulation of the immune system, with increased type I IFN levels and higher CD8^+^ T-cell tumor infiltration. Importantly, the treatment was well tolerated [88].

The antitumor properties of TLR*ag* are also being explored in the field of CAR-T cells (Figure 3). Some researchers are engineering the chimeric antigen receptor to induce immunostimulatory elements of the TLR pathways, such as MyD88 or the TIR domain [89]. Other groups have explored combinations of TLR*ag* with CAR-T cell therapy. For example, Luo et al. described the ability of a folate-targeted TLR7*ag* (FA-TLR7-1A) to specifically reactivate TAMs and MDSCs. They described that FA-TLR7-1A significantly augmented the efficacy of CAR-T cell therapy in the breast cancer 4T1 model, through re-polarization of TAMs/MDSCs from an M2-like anti-inflammatory to M1-like pro-inflammatory phenotype [90]. In another study, i.t. administration of both poly(I:C) and CpG*nt* was used as adjuvant for adoptive T-cell therapy in an established model of melanoma. The treatment activated host DCs and enhanced antigen cross-presentation with adoptively transferred T cells, improving their antitumor activity via an IFN-γ-dependent mechanism [91].

### 4.4. Combination of TLR Agonists and Radio-/Chemo-Therapy

Chemotherapy and radiotherapy (RT) continue to be the main therapeutic approaches for the treatment of solid tumors (Figure 3). Following the outbreak of immunotherapy as a highly effective approach in some patients, many studies are being undertaken to combine cytotoxic drugs and/or RT with immunotherapy. As an example, the combination between platinum-based chemotherapy and ICI is now used as a first-line therapeutic option in patients with NSCLC [92]. Several preclinical studies have also explored the combination of endosomal TLR*ag* with chemotherapy. For instance, Johnson et al. showed that i.t. injection of resiquimod and paclitaxel using a complex NP-formulation led to cures and reduction of toxicity compared to the free drugs in the CT26 colon cancer model [93]. Seth et al. found that poly γ-glutamic acid-based combination of water-insoluble paclitaxel and imiquimod injected i.t. in a mouse melanoma tumor model resulted in drastic inhibition of tumor growth [94]. In the CT26 model, TLR9 activation acts as a sensor for tumor-released DNA to modulate antitumor immunity after chemotherapy [95]. Such an adjuvant effect of chemotherapy was mediated by the release of tumor DNA, which caused antigen uptake and maturation of DCs within the tumor. TLR*ag* + chemotherapy combination has also been tested in clinical trials. Ferris et al. described the addition of motolimod (TLR8*ag*) to standard combination chemotherapy and cetuximab in patients with HNSCC. They found that this regimen was well tolerated but did not improve PFS or OS. However, significant benefit was observed in HPV^+^ patients and those with injection-site reactions, suggesting that TLR8 stimulation may benefit particular groups of patients [96].

RT is one of the main inducers of immunogenic cell death (ICD), releasing DAMPs (e.g., dsRNA or tumor antigens) into the TME. Recent advances in the field, allowing for a better control of dose, time, and localization of RT [97], have proven that this therapeutic modality may elicit an efficacious antitumoral immune response. In addition, DNA fragments released after irradiation can activate the cGAS/STING pathway, resulting in the production of type I IFN and T-cell cross-priming [98,99]. However, RT response is at times limited, and TLR*ag* have been combined with RT to boost activity of DCs and improve the priming of naive T cells [100]. For example, in murine models of lymphoma, combination of RT with resiquimod increased the number of cytotoxic T cells leading to tumor control [101]. Tumor regressions were observed in colorectal cancer models when the TLR7*ag* DSR-29133 was used in combination with fractionated RT [102]. Combined therapy was curative in a high proportion of mice bearing CT26 tumors and was dependent on the activity of CD8^+^ T lymphocytes, but independent of CD4^+^ T-cells and NK cells [102]. In the Lewis lung carcinoma model, combination of RT with a TLR9*ag* reduced tumor growth and metastasis in both wild type and B-cell(^-^/^-^) mice [103]. This study also revealed an increased humoral response and higher numbers of NK in the TME. Several clinical trials assessing the efficacy of TLR*ag* in combination with RT have been published, and some others are currently ongoing. A phase I trial using poly-ICLC in combination with low doses of fractionated RT found an improvement in PFS and OS in a small cohort of hepatocarcinoma patients not eligible for surgery or liver transplant [104]. A multicentric Phase I/II trial has evaluated the combination of i.t.-injected SD-101 (TLR9*ag*) and low-dose RT in patients with untreated indolent lymphoma. Results showed favorable outcomes and the combination was well tolerated. After treatment, an increase in the number of CD8^+^ and CD4^+^ effector T cells was observed, while the number of Tregs was diminished [105]. Our institution (University of Navarra, Pamplona, Spain) has recently registered a Phase I/II clinical trial aiming to study the efficacy of BO-112 (TLR3*ag)* in combination with stereotactic body radiation therapy (SBRT) in PD-1/PD-L1 refractory metastatic NSCLC patients (NCT05265650).

### 4.5. Combination of TLR Agonists with Other Therapeutic Agents (Genetic, Epigenetic, Metabolic Targets)

In the last decades, a great number of genetic, epigenetic, and metabolic alterations have been identified in tumors. These modifications are of interest in the context of personalized medicine, and targeted therapeutic approaches have been developed for intervention in the clinical practice [106]. Because most targeted therapies generate resistance through different mechanisms, combination approaches with other strategies have been tested to obtain maximal therapeutic benefit (Figure 3). In the context of TLR*ag*, several studies have assessed combinations with targeted therapy. As an example, Levy and colleagues demonstrated that CpG*nt* increased the efficacy of ibrutinib (small drug targeting Bruton’s tyrosine kinase) in various subcutaneous B-cell lymphoma models (H11, A20, BL3750) [107]. The combination of both agents resulted in a T-cell dependent eradication of the treated tumor. A partial response was also observed in the non-treated tumors, and these effects were abolished with the depletion of CD4^+^ and/or CD8^+^ T cells. In similar ways, systemic administration of the growth factor FMS-like tyrosine kinase 3 ligand (FLT3L) in murine melanoma models expanded the population of CD103^+^ dendritic cells in the TME, “preparing” them for the activation with poly(I:C). Furthermore, this approach demonstrated a better response to the subsequent administration of BRAF inhibitors or anti-PD-1 in the aforementioned models [108]. Similarly, TLR*ag* also increased the activity of monoclonal antibodies targeting HER2 in triple negative breast cancer (TNBC), as Charlebois and colleagues demonstrated with the combination of poly(I:C) and CpG*nt* together with ErbB2 blockade in murine TNBC models [109]. Targeted therapy and TLR*ag* have also been tested in clinical trials. Smith et al. described the safety and beneficial therapeutic effect of IMO-2055 (TLR9*ag*) in combination with erlotinib and bevacizumab in advanced NSCLC. They found that the treatment was well tolerated and that 76% of the patients showed stable disease [110].

Another approach consisted of the combination of TLR*ag* with drugs that modify the metabolism in the TME. Seth et al. used the vasculature disrupting agent 5,6-dimethylxanthenone-4-acetic acid (DMXAA) in combination with PLGA-NPs loaded with gardiquimod (TLR7/8*ag*) to induce tumor regression and increase the survival of murine melanoma models [111]. Another study described the combination of TLR*ag* with targeted therapies directed towards inhibitory receptors on myeloid cells, such as CD200R [112]. I.t. administration of resiquimod inhibited the growth of CT26 colon carcinoma and decreased CD200R expression in tumor-infiltrating immune cells. Cured mice were resistant to re-challenge. The treatment changed the phenotype of myeloid cells, as infiltration with immature MHC-II^+^ macrophages decreased and, in parallel, monocytes and immature MHC-II^-^ macrophages increased. Interestingly, CD11b^+^ cells from cured mice adoptively transferred in naïve mice were protective of tumor growth [112].

Alternative combination approaches included electroporation, phototherapy, or oncolytic viral therapy to increase the release of tumoral neoantigens [113]. For instance, 1V270 (TLR7*ag*) or anti-PD-1 following irreversible electroporation (IRE) in a subcutaneous model of pancreatic cancer (KPC4580P) led to an increased antitumoral response [114]. Similarly, IRE in combination with poly-ICLC in both subcutaneous murine and orthotopic rabbit models of liver cancer resulted in therapeutic improvement and growth inhibition of the untreated tumors [115].

## 5. Conclusions and Future Perspectives

Intratumoral injection of endosomal TLR*ag* has proven to trigger innate and adaptive immune responses, which results many times in striking antitumor effects (even in tumor rejections) in a large number of cancer experimental models. Acting as vaccine adjuvants or direct antitumor agents, mainly in combination with different therapies, TLR*ag* hold great promise in the new era of cancer immunotherapy. The initial failure of these drugs in clinical trials, where high toxicity and limited efficacy was observed, is likely to be changed in the near future. Key aspects to overcome these limitations will include: (a) intratumor administration, unlike the former trials where limited antitumoral activity and immunotoxicity were due to systemic administration; (b) conjugation or encapsulation in NP-formulations that allow accumulation within the tumor and controlled release of the drug towards the immune cells; (c) combination strategies with novel immunotherapy agents, other TLR*ag*, STING*ag*, fractionated radiotherapy, or other treatment modalities under investigation, either in preclinical experiments or clinical trials. In conclusion, the efforts to find out the best strategy for the use of TLR*ag* is likely to render a new paradigm for the efficacious treatment of many types of cancer.

## Figures and Tables

**Table 1:** Main endosomal TLR agonists, biological and antitumor effects, and examples of combinations of TLR-TLR*ag* and TLR-STING*ag*.

TLR	Ligand	Cancer and Model	Observations	References
TLR3	Poly(I:C)	Syngeneic animal models and clinical trials	High antitumoral efficacy in several preclinical models; clinical trials were not successful	[54,55,62,66,67]
	Poly-ICLC (hiltonol^®^)	Syngeneic animal models and clinical trials	Pharmaceutical formulation is more stable than poly(I:C) and more effective but highly toxic	[53]
	Poly(A:U)	B16.F10-OVA melanoma murine model	Antitumoral efficacy, activation of DCs, increase in CD8^+^ T cell infiltration, and decrease in IL-10-producing M2-like macrophages	[56]
TLR7/8	R837 (imiquimod^®^)	FDA-approved for the treatment of basal cell carcinomas	Promotes apoptosis and cell-mediated antitumor immunity	[9,64,67]
	R848 (resiquimod^®^)	MC38 colon cancer and B16.F10 melanoma murine models, orthotopic model of NSCLC	Complete tumor regression, preventing tumor growth after re-challenge	[58,59]
		Clinical trials in hematological neoplasias and solid tumors	Controversial results related to poor antitumoral activity and immunotoxic effects	[42], reviewed in [47]
	1V199, 1V270	B16cOVA murine model	Inhibition of tumor growth when low repeated doses were used	[61]
TLR9	CpG*nt*	Syngeneic animal models and clinical trials	Activate pDCs and CTLs, enhancing T cell-mediated antitumor immunity; in clinical trials, short half-life in serum leading to low activation of NK cells and CTLs, and increase of pro-inflammatory cytokine production	[62], Reviewed in [45]
TLR3 + TLR7/8	Poly(I:C) + R848	Lung adenocarcinoma and fibrosarcoma murine models	Antitumoral activity mainly driven by macrophage reprogramming, which promoted the activation of innate and adaptive immune responses against the cancer cells	[63]
		Lymphoma murine models	Profound antitumor effects in the context of peptide vaccination	[73]
	Poly(I:C) + R837	B16.F10(OVA) melanoma murine model	Synergistic activation of antitumor immune responses and direct killing of cancer cells in established tumors	[72]
TLR3 + TLR9	Poly(I:C) + CpG*nt*	Murine glioma model	Inhibition of tumor growth and improved median survival, by activation of an antitumor phenotype of microglia	[68]
TLR7/8 + TLR9	3M-052 + CpG*nt*	Colon carcinoma murine model	Upregulation of Th1 cytokine-expression, reduction in the number of tumor resident MDSCs, increasing in the accumulation of NK cells and CD8^+^ T lymphocytes, leading to strong and long-lasting antitumoral immune responses	[69]
TLR4 + TLR7/8	HMGB1 + R848	CT26 murine tumor model	Increased the infiltration of T cells and activation and homing of tumor-infiltrating DCs to the draining lymph node, eradication of large established tumors and resistance to re-challenge	[70]
TLR2/6 + TLR 7/8	Pam2CSK4C + azide	B16.F10 melanoma murine model	CD8^+^ T cell and NK cell antitumor responses, inhibits tumor growth and reduced adverse effects	[71]
TLRs + STING agonists	CpG*nt* + cGAMP	EG-7 and B16 F10 murine tumor models	Synergistic activation of NK cells, resulting in high production of IFN-γ and activation of CD8+ T cell response in vivo	[74]
	Poly(I:C)-nanocomplex (BO-112^®^) + DMXAA	Colon cancer and melanoma murine models	Strong antitumoral activity and abscopal effect, while none of the single drugs showed such an activity	[75]

## Data Availability

Non-applicable.
